# Sunflower Oil–Beeswax Oleogels and Bigels Enriched With Quercetin‐Rich Onion Phenolics for Biscuit Fat Replacement: Physicochemical, Antioxidant, and Stability Outcomes

**DOI:** 10.1002/fsn3.71423

**Published:** 2026-01-07

**Authors:** Mahmoud Younis, Mohamed Abdelbaset Salama, Diaeldin Omer Abdelkarim, Reham M. Kamel, Said El Harkaoui, Mostafa Ali, Eslam Elshaqwir, Mohamed Abdin, Mohamed El‐Bana, Mona Hussein Hassan, Maha Gomaa, Mohamed Saleh, Amira H. Sharshar, Amira E. Abd El‐Gwad, Menna Allah M. Youssef, Shimaa E. Abd EL‐Hamed, Alshaimaa M. Hamouda, Naglaa K. Beltagy, Tahia R. Al‐Khawaga, Mariam A. El‐Khatib, Nahla A. Felays, Mahmoud Elsayed

**Affiliations:** ^1^ Chair of Dates Industry and Technology King Saud University Riyadh Saudi Arabia; ^2^ Agricultural Research Center Food Technology Research Institute Giza Egypt; ^3^ Department of Agricultural Engineering, Faculty of Engineering University of Khartoum Khartoum Sudan; ^4^ Agricultural Engineering Research Institute, Agricultural Research Center Giza Egypt; ^5^ Max Rubner‐Institut (MRI), Department of Safety and Quality of Cereals, Working Group for Lipid Research Detmold Germany; ^6^ Faculty of Agriculture, Kafrelsheikh University Kafrelsheikh Egypt; ^7^ Food Science and Technology Department, Faculty of Agriculture Tanta University Tanta Egypt

**Keywords:** antioxidant activity, bigel, fat replacement, oleogel, phenolic extract

## Abstract

This study evaluated sunflower oil–beeswax oleogels (OLGL) and bigels (BGL) as fat replacers in biscuits and introduced a phenolic‐enriched bigel (BGLX) using a quercetin‐rich onion extract. Converting the oleogel to a bigel softened the network and lowered thermal transitions (firmness: 4.37–3.77 N; melting point: 55.4°C–48.4°C for OLGL and BGL, respectively), with only minor, non‐significant additional softening in BGLX. Oil binding capacity decreased relative to OLGL (78.35%) in bigels (73.17% BGL; 72.57% BGLX). Phenolic enrichment increased antioxidant capacity in gels (TPC: 3.95–5.62 mg GAE/g; DPPH: 28.15%–39.73% from sunflower oil to BGLX, respectively) and raised thermal resistance (DSC Td: ~229°C to ~248°C for BGL and BGLX, respectively). In biscuits, structured lipids maintained processing functionality and increased moisture retention, with protein and ash unchanged. At Day 0, oxidative status was similar among structured‐lipid biscuits; during 90‐day ambient storage, peroxide growth was attenuated stepwise and remained within Codex limits (control 8.07 vs. BGLX 4.54 meq O_2_/kg). AFM/SEM showed smoother networks in OLGL and rougher, more complex features in bigels, consistent with dual‐phase structuring. Practically, bigels enable lower‐saturated‐fat bakery reformulation, and phenolic enrichment offers an additional, clean‐label route to oxidative stability without penalizing texture.

## Graphical abstract

Sunflower oil–beeswax bigels delivered oleogel‐like functionality while softening the network and modestly lowering melting behavior; phenolic enrichment with a quercetin‐rich onion extract increased antioxidant capacity without altering mechanics. As a 1:1 fat replacer in biscuits, bigels raised moisture retention and maintained texture, and the phenolic‐enriched bigel further attenuated peroxide formation over 90‐day storage within Codex limits. These clean‐label systems support lower‐saturated‐fat bakery reformulation with improved oxidative stability.

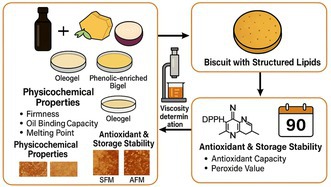

## 1 Introduction

High intake of trans and saturated fats has been associated with elevated risks of cardiovascular disease, type 2 diabetes, inflammation, and metabolic syndrome (López‐Pedrouso et al. 2021; Roche 2005). In 2015, the U.S. Food and Drug Administration (FDA) ruled that partially hydrogenated oils are no longer Generally Recognized as Safe (GRAS), and the European Food Safety Authority (EFSA) later confirmed the clear link between trans‐fat consumption and coronary heart disease. These regulatory actions have accelerated efforts to reformulate foods with healthier lipid alternatives that can provide technological functionality while improving nutritional profiles.

Structured lipid systems such as oleogels and bigels have gained attention as promising fat mimetics. Oleogels are viscoelastic three‐dimensional gels created by structuring edible oils with gelators such as waxes, mono‐ and diglycerides, or phytosterols (Blach et al. 2016; Manzoor et al. 2022). Bigels, in turn, are biphasic systems combining oleogel and hydrogel networks, enabling higher stability and the simultaneous delivery of lipophilic and hydrophilic compounds (Shakeel et al. 2019; Martín‐Illana et al. 2022). Their dual‐network structure reduces oil leakage, enhances texture, and opens opportunities for functional ingredient delivery.

Sunflower oil is particularly attractive for oleogel and bigel development because of its high global availability, favorable fatty acid profile (60%–70% linoleic acid, 20%–30% oleic acid), and oxidative stability (Nid Ahmed et al. 2024). With global production exceeding 20 million metric tons in 2022/2023, sunflower oil is both nutritionally advantageous and economically feasible for large‐scale applications. Its unsaturated fatty acid profile and low peroxide values make it a suitable base oil for replacing saturated fats in processed foods.

A major advantage of oleogels and bigels is their ability to incorporate natural antioxidants, improving oxidative stability while delivering health‐promoting compounds. Rather than highlighting generic examples such as fish coatings, a more relevant case is the use of antioxidant‐enriched oleogels as edible coatings for bread, which significantly improved oxidative stability and extended shelf life. This demonstrates their dual role as both fat replacers and carriers of bioactive compounds that protect food matrices against deterioration.

Recent bakery applications further support this potential: sunflower oil‐based oleogels have successfully replaced butter in muffins without compromising texture (Espert et al. 2023), while bigels have maintained crispness and shelf life in breadsticks (Nutter et al. 2023). These findings show that structured lipids can reduce saturated fat while preserving consumer‐acceptable quality in baked goods.

Therefore, the present study aimed to formulate sunflower oil–beeswax oleogels and bigels, including a phenolic‐enriched bigel (BGLX) prepared with quercetin‐rich onion extract. This work focused on characterizing the physicochemical, structural, thermal, and antioxidant properties of the gels and evaluating their application as fat replacers in biscuits. Particular attention was given to moisture retention, texture, phenolic content, and oxidative stability during 90 days of storage, thereby providing new insights into the use of phenolic‐enriched bigels in bakery reformulation.

## 2 Materials and Methods

### 2.1 Materials

**FIGURE 1:**
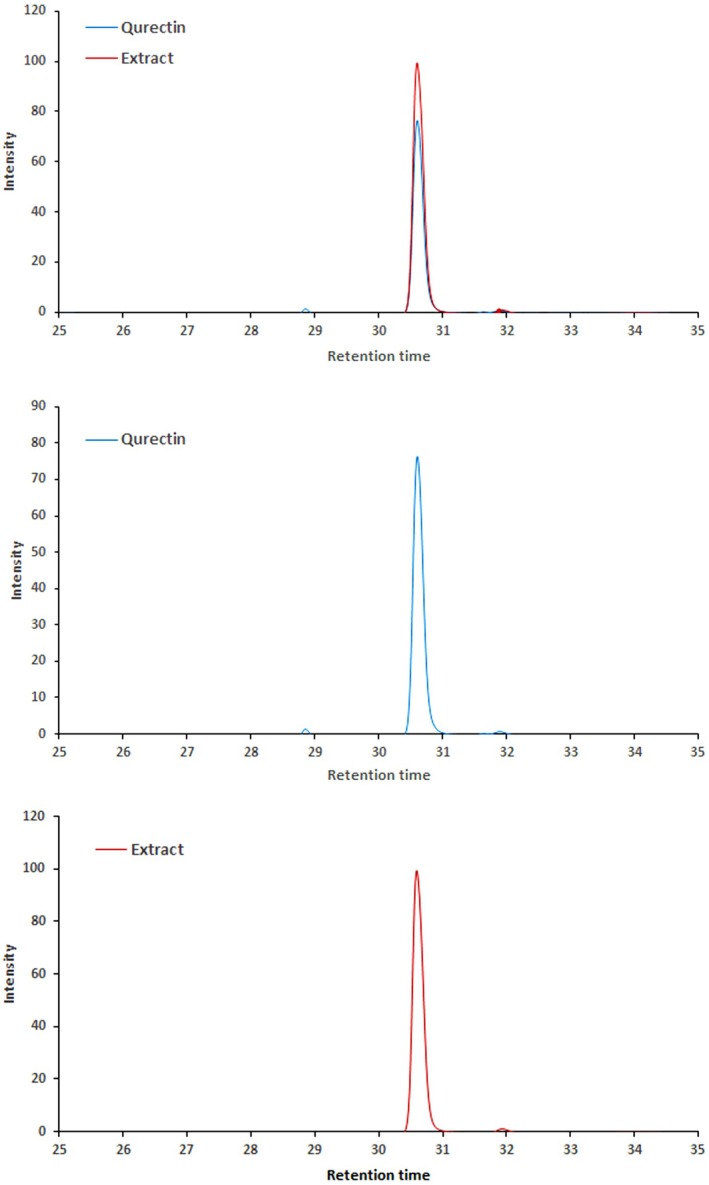
Onion extract powder rich with quercetin.

Onion extract powder rich in quercetin ( *Allium cepa* L) was purchased from Rudolf Wild GmbH & Company KG. This extract was incorporated into the BGLX bigel formulation as a source of phenolic antioxidants. According to the supplier, the extract contained 95.49% ± 1.5% quercetin (dry weight basis), which was confirmed in our lab using HPLC‐DAD analysis. This extract was incorporated into the BGLX formulation (0.25 g per batch) to evaluate its antioxidant effect in the structured lipid system (Figure 1). Beeswax (BW) was supplied by Kahlwax Co. (Kalh GmbH & Co., Trittau, Germany). All standards, chemicals, and solvents utilized in the study were of analytical grade, procured from Sigma Chemical Co. (St. Louis, MO, USA) and Merck (Darmstadt, Germany). Sunflower oil (high‐oleic; acidity ≤ 0.2% as oleic acid; PV < 5 meq O_2_/kg) was produced in Dec‐2024 and stored at 4°C ± 1°C in the dark until use. Sunflower oil was sourced from Tanta Company of Oils and Soaps, located in Tanta City, Egypt. The oil was produced in December 2024. It was supplied in dark amber glass bottles (500 mL) to minimize light exposure and oxidative degradation. The oil was stored at 4 ± 1 in the dark until use. This high‐oleic variant was selected for its enhanced oxidative stability and health‐promoting fatty acid profile, making it suitable for oleogel and bigel formation.

### 2.2 Methods

#### 2.2.1 Oleogel and Bigel Preparation

According to bigels phase composition and preparation route, the bigels obtained in this study can be classified as W/O‐type food‐grade bigels, in which the beeswax‐structured sunflower oleogel constitutes the continuous lipid network, while the gelatin–glycerol hydrogel is present as discrete aqueous domains dispersed within this network (oil: water = 60:40, w/w). This structural arrangement is consistent with oleogel‐rich bigel systems prepared with oil‐soluble surfactants, as reported for similar food‐grade bigels. For clarity, the following abbreviations were used throughout: OLGL (sunflower oil–beeswax oleogel), BGL (bigel without phenolic extract), and BGLX (bigel enriched with a quercetin‐rich onion phenolic extract).

To prepare the oleogel (OLGL), 8 g of beeswax (8 g BW + 92 g oil per 100 g batch) was melted in a glass beaker at 80°C for 7 min. Sunflower oil was then added, and the mixture was heated to 80°C for 20 min while stirring at 700 rpm using a magnetic stirrer (Salama et al. 2024). For the bigel without extract (BGL), the method of Rehman et al. (2014) was used with some modifications. The previously melted 8 g of beeswax was combined with the oil and Tween 80 (1% w/w of oil). Gelatin (0.5%) and glycerol (1.5%) were dissolved in distilled water to form hydrogel. All bigels were prepared using a gravimetric oil: water ratio of 60:40 (w/w), 120 g oleogel: 80 g hydrogel. The two mixtures were emulsified together using an Ultra‐Turrax T25 homogenizer (IKA‐ Staufen, Germany) at 18,000 rpm for 5 min at room temperature (25°C ± 2°C). For the bigel with extract (BGLX), 0.25 g of onion extract powder was dispersed into the hydrogel phase (80 g) before emulsification with the oil–beeswax mixture. This corresponds to 0.3125% (w/w) relative to the hydrogel and 0.125% (w/w) relative to the total bigel (0.25 g per 200 g batch). This phenolic‐rich formulation was designed to enhance the antioxidant potential of the bigel system (Keskin Uslu and Yılmaz 2021). The resulting mixtures were left at room temperature overnight to allow for gelation and then stored at 4°C in the refrigerator until analysis.

##### 2.2.1.1 Gelation Time

The samples (10 mL) in glass tubes (inner diameter 16 mm, length 100 mm), giving an analyzed gel column thickness (height) of ~5.0 cm, were fully melted in a water bath at 90°C and maintained for 20 min to allow for isothermal setting. Room‐temperature equilibration of the sample core was verified in a parallel, identically filled tube using a K‐type micro‐thermocouple positioned at mid‐height; the timer started when the core reached 22.0°C ± 0.5°C. Afterward, the tubes were removed from the water bath and brought to room temperature, at which point a timer was started. The gelation time was recorded when the tubes were tilted 90° and no flow of the organogel was observed (Yılmaz and Öğütcü 2015).

##### 2.2.1.2 Oil Binding Capacity (OBC)

1 mL of sample was transferred into a pre‐weighed Eppendorf tube (a) and then placed in a refrigerator at 4°C for 1 h. After refrigeration, the tube was weighed again (b). The tube was then centrifuged at 9167 *g* for 15 min at room temperature (25°C). Following centrifugation, the tube was inverted onto a paper cloth to drain any excess liquid oil. After the drainage process, the tube was weighed once more (c) (Yılmaz and Öğütcü 2015). OBC values were subsequently calculated using the specified equation.

$$\%\text{Released oil}=\frac{\left(b-a\right)-\left(c-a\right)}{\left(b-a\right)}\times 100$$

%OBC=100−%Released oil

##### 2.2.1.3 Color Determination

Measured with Minolta CR‐400 under D65 illuminant, 10° standard observer, 8 mm aperture, on samples equilibrated to 22°C ± 2°C. The instrument was calibrated against a certified white tile before each session.

Whiteness was calculated as $\begin{array}{c} {\text{WI}}_{\text{CIE}}=100- \end{array}$ $\begin{array}{c} \sqrt{\left(100-L*\right)+\left(a*\right)2+\left(b*\right)2} \end{array}$. Yellowness was calculated as YI_Lab_ = 142.86 × b*/L*.

##### 2.2.1.4 The Centrifuge Stability Test

The centrifuge stability test was performed by subjecting a 5 g sample in tubes to a centrifugation force of 1300× *g* for 15 min at room temperature. The stability of the gel was then assessed through visual inspection (Yilmaz and Demirci 2021).

##### 2.2.1.5 Melting Point

In summary, 7 g of molten sample was placed into glass tubes. The tubes were then immersed in a water bath set to 38°C and heated at a constant rate of 1°C every 3 min until the samples were completely melted and became transparent liquids (Zampouni et al. 2022).

##### 2.2.1.6 Firmness

The firmness was assessed using a Texture Analyzer (CT3 4500, Brookfield, USA) at room temperature. The samples (30 g) were stored in a 50 mL glass beaker in a refrigerator at 5°C for 24 h. Each sample was then removed and tested individually, with the probe penetrating at a speed of 0.2 mm/s to a depth of 5 mm. The firmness was determined by recording the maximum penetration force (Öǧütcü and Yılmaz 2014).

##### 2.2.1.7 Free Fatty Acids and Peroxide Values

The free fatty acid content was determined using the titration method as described in AOCS (2017). Approximately 5 g of the sample was weighed into a 250 mL Erlenmeyer flask and dissolved in 50 mL of a neutral solvent mixture consisting of ethanol and diethyl ether (1:1, v/v) that had been previously neutralized with 0.1 N NaOH. Two to three drops of phenolphthalein indicator were added, and the mixture was titrated with 0.1 N NaOH until a persistent faint pink color appeared. The FFA content was calculated as percent oleic acid.

Peroxide value was measured following AOCS (2017). 5 g of the sample was weighed into a 250 mL glass‐stoppered Erlenmeyer flask, to which 30 mL of a chloroform–acetic acid mixture (2:3 v/v) was added. The flask was gently swirled to dissolve the sample. Then, 0.5 mL of saturated potassium iodide (KI) solution was added, and the mixture was kept in the dark for 1 min. Afterward, 30 mL of distilled water was added. The liberated iodine was titrated with 0.01 N sodium thiosulfate (Na_2_S_2_O_3_) using starch solution as an indicator.

##### 2.2.1.8 Phenolic Extraction

For this extraction, 50 g of each sample (oil‐based matrices—sunflower oil (SO), the oleogel (OLGL), and the lipid phase of the bigels (BGL, BGLX)) were processed using a glass column filled with silica gel (60 Å pore size). The column was first conditioned with a hexane–methanol mixture (1:1 v/v), followed by rinsing with a hexane–ethyl acetate mixture (9:1 v/v) to prepare it for sample loading. The oil sample, dissolved in hexane, was then applied to the column, allowing the phenolic compounds to be eluted with methanol. The collected extract was concentrated by evaporating the solvent under vacuum at 40°C. To prevent oxidation, the resulting extracts were flushed with nitrogen and stored at −20°C (Steel et al. 2005). For biscuits, a ground sample (10.0 g) was extracted with 50 mL 70% ethanol (ultrasonication 30 min, 22°C; centrifugation 4000 × g, 10 min); the supernatant was used for TPC and DPPH determinations (no silica‐gel step).

##### 2.2.1.9 Total Phenolic Content (TPC)

The total phenolic content (TPC) of gel and biscuit samples was determined using the Folin–Ciocalteu colorimetric method, following the protocol of with slight modifications. Briefly, 100 μL of the sample extract or gallic acid standard (0–200 mg/L) was mixed with 750 μL of 10% (v/v) Folin–Ciocalteu reagent. After standing for 5 min, 750 μL of 7.5% (w/v) sodium carbonate solution was added. The mixture was then incubated in the dark at room temperature (22°C ± 2°C) for 45 min. Absorbance was measured at 765 nm using a UV–Vis spectrophotometer (Analytik Jena Specord 250). A calibration curve was generated using gallic acid, and the results were expressed as mg gallic acid equivalents per gram of sample (mg GAE/g). All measurements were performed in triplicate (Alshehri et al. 2025).

##### 2.2.1.10 Antioxidant Activity Using DPPH Assay

The antioxidant activity of the samples was determined by the DPPH radical scavenging method. In brief, 0.1 mL of sample extract was mixed with 2 mL of freshly prepared 0.2 mM DPPH solution in methanol. A blank (methanol only) and a control (DPPH without sample) were prepared simultaneously. All mixtures were kept in the dark at room temperature (22°C ± 2°C) for 30 min. The absorbance was measured at 517 nm using a UV–Vis spectrophotometer (Analytik Jena Specord 250) (Alshehri et al. 2025). The DPPH radical scavenging activity was determined using the provided equation:

$$\begin{array}{c} \text{DPPH antioxidant activity}\;\left(\%\right)\\ \;=\left(\left(\text{control}\;\mathrm{ab}-\text{sample}\;\mathrm{ab}\right)/\left(\text{control}\;\mathrm{ab}\right)\right)\times 100 \end{array}$$

where: control ab represented the absorbance of the DPPH working solution without the sample, and sample ab corresponded to the absorbance of the DPPH working solution when mixed with samples.

##### 2.2.1.11 Scanning Electron Microscopy (SEM) and Atomic Force Microscopy (AFM)

For SEM, the samples were mounted onto aluminum sample holders and coated with a 10 nm layer of gold using a Sputter Coater 108 auto (Cressington Scientific Instruments, Watford, UK). The aluminum holder was then placed in the SEM unit (EVO 40XVP, Carl Zeiss, Milan, Italy), which operated at room temperature under vacuum conditions. Imaging was performed with an acceleration voltage of 20 kV, and the images were captured using SmartSEM v. 5.09 software (Carl Zeiss, Milan, Italy). SEM images were acquired at 800× (accelerating voltage 20 kV, WD 12 mm; scale bar = 15 μm). AFM: tapping mode, 20 × 20 μm scan area (512 × 512 pixels); Z‐scale indicated on each panel.

For AFM sample preparation, oleogel and bigel samples were gently melted at 70°C–80°C (10 min) to obtain pourable fluids. Using a heated micropipette tip, 20 μL of molten sample was dispensed onto freshly cleaved mica (V‐1 quality) pre‐warmed to 40°C. Films were formed by doctor‐blading the drop with a fixed 100 μm gap applicator to yield a uniform wet layer; the substrates were then equilibrated at 22°C ± 2°C for 30 min to allow gel setting. For bigels, the same protocol was applied; excess surface moisture was wicked from the film edge with lint‐free paper immediately after drawdown to avoid meniscus artifacts. Surface topography of the films was examined using a NanoScope MultiMode Atomic Force Microscope (AFM) from Veeco Metrology Inc. (Santa Barbara, USA) equipped with a scanning probe microscopy (SPM) probe (Abdin et al. 2024).

##### 2.2.1.12 Differential Scanning Calorimetry (DSC)

Thermal behavior was measured on a DSC 200 (NETZSCH, Germany) using a two‐cycle protocol to resolve glass/softening transitions. Approximately 2–4 mg of sample was sealed in aluminum pans (pin‐holed lids for gels). The instrument was temperature and enthalpy calibrated with indium. Each sample underwent: (1) thermal‐history erase: heat to 90°C at 10°C·min^−1^; (2) controlled cooling to 0°C at 10°C·min^−1^; 5 min isotherm; (3) reheating from 0°C to 200°C at 5°C·min^−1^ under N_2_ (20 mL·min^−1^).

Tg was determined on the reheating scan as the midpoint of the step change in heat capacity (Cp), constructed by tangents to the pre‐ and post‐transition baselines (ASTM E1356). Melting parameters were taken as *T*_m,onset_, *T*_m,peak_, and ΔH_m_ (area of the endotherm, J·g^−1^). A high‐temperature endothermic event was also reported as *T*_d,onset_ and *T*_d,peak_ when present.

##### 2.2.1.13 Viscosity Determination

The viscosity of the samples was determined using a Viscometer (LR Lamy Rheology instrument). The samples were first melted in a water bath at 80°C. Once fully melted, the oleogel samples were transferred to the viscometer's sample cell, and their viscosity was recorded as the temperature gradually decreased from 80°C to 20°C at a rate of approximately 1°C per minute. The viscosity values were then plotted against the corresponding temperatures (Sahu et al. 2020).

#### 2.2.2 Biscuit Preparation

The method of Adeyemo et al. (2022) was employed with moderate modification. For each batch, biscuits were prepared using the following base formula per batch: wheat flour (200 g), fat (80 g), granulated sugar (60 g), water (120 mL), baking powder (4 g), salt (1 g), vanilla (1 g). Fat (control: margarine; treatments: oleogel or bigels, 100% fat replacement) was added to the flour (200 g); water (120 mL) was incorporated and adjusted as needed to reach the target dough moisture before mixing to a smooth dough. The dough was rolled out by hand on a tray (5 mm height) and was cut with the aid of a rectangle‐shaped biscuit cutter (4.9 cm diameter). The dough pieces neatly were arranged on a baking tray laced with buttered aluminum foil were baked (160°C ± 2°C for 30 min) in a pre‐heated Unox steam convection oven and cooled to room temperature for 30 min. Biscuits were packed in airtight, thick, zip‐seal food bags, placed in low‐density polyethylene bags, and preserved at 4°C ± 2°C. Control biscuit samples were prepared with margarine as the sole fat (no oleogel/bigel). For treatments, margarine was completely (100%) replaced with OLGL, BGL, or BGLX.

##### 2.2.2.1 Chemical Composition of Prepared Biscuits

Moisture, ash, crude protein, fiber, and fat were determined according to AOAC (2016). Carbohydrates were calculated by difference as follows: available carbohydrates = [100 − (%fat − %proteins − % fiber − %ash)].

##### 2.2.2.2 Storage of Prepared Biscuits

###### 2.2.2.2.1 Packaging and Storage Conditions

The freshly baked biscuits were cooled at room temperature (22°C ± 2°C) for 1 h before packaging. Biscuits were packed in transparent, food‐grade low‐density polyethylene bags with each bag containing 10 biscuits (approximately 30 g each). Packaging was carried out immediately after cooling to minimize moisture uptake and oxidative changes. The packaged samples were stored at ambient room temperature (22°C ± 2°C) for a period of 90 days in a dry, dark environment. Moisture content, texture analysis (hardness), and peroxide value were assessed at regular intervals (0, 30, 60, and 90 days) to monitor biscuit stability. Analytical evaluations were initiated within 24 h of packaging and conducted in triplicate, with average values reported. At each designated time point (0, 30, 60, and 90 days), biscuit samples (3 pieces per replicate) were homogenized using a laboratory blender to ensure uniformity.

###### 2.2.2.2.2 Moisture Content

Moisture content was determined gravimetrically according to AOAC (2016). Approximately 5 g of ground biscuit sample was weighed into a moisture dish and dried in a hot air oven at 105°C until a constant weight was achieved. The difference in weight was recorded and expressed as percentage moisture content.

###### 2.2.2.2.3 Peroxide Value

Peroxide value (PV) was determined using the same method described for oleogels (AOCS 2017). Briefly, 5 g of the ground biscuit sample was mixed with 30 mL of chloroform–acetic acid (2:3, v/v) and processed as described previously. The sample was titrated with 0.01 N sodium thiosulfate in the presence of starch indicator, and PV was expressed as meq O_2_/kg of lipid extracted from biscuits.

###### 2.2.2.2.4 Texture Analyses

The texture of the sunflower oil–beeswax oleogel and bigels was evaluated by texture profile analysis (TPA) using a texture analyzer (CT3‐4500, Brookfield, Middleboro, MA, USA) equipped with a 50 N load cell. Samples were poured into cylindrical containers and stored under refrigerated conditions until analysis, then tempered to 25°C ± 1°C. A cylindrical stainless‐steel probe (⌀ 10 mm) was used to perform a two‐cycle compression test. The probe was driven vertically into the sample to a deformation of 50% of its original height at a constant speed of 1.0 mm/s, with a waiting time of 5 s between the first and second compression. From the force–time curves, hardness (N, cycle 1) was defined as the maximum force of the first compression, adhesiveness (mJ) as the negative area under the curve during probe withdrawal after the first cycle, cohesiveness as the ratio of the positive area under the second compression curve to that of the first compression, and springiness (mm) as the distance recovered by the sample between the end of the first compression and the start of the second. All measurements were performed in triplicate for each formulation and results were expressed as mean ± standard deviation.

##### 2.2.2.3 Statistical Analysis

The data were analyzed using SPSS software (version 16.0, SPSS Inc., Chicago, IL, USA). One‐way analysis of variance (ANOVA) was applied to determine statistically significant differences among the samples. Tukey's test was used for comparing between means. Results were expressed as mean ± standard deviation based on three replicates, and differences were considered statistically significant at *p* < 0.05.

## 3 Results and Discussion

### 3.1 Physical and Chemical Properties of Sunflower‐Oil Oleogel and Bigel Systems

The physico‐chemical properties of sunflower oil, OLGL, BGL, and BGLX demonstrated distinct variations, particularly in gelation time, oil binding capacity, firmness, melting point, and oxidative stability indicators (FFA and PV) (Table 1).

**TABLE 1:** Physico‐chemical, antioxidant, color properties and sensory evaluation of sunflower oil‐beeswax oleogel and bigel.

Properties	Sunflower oil	OLGL	BGL	BGLX
Gelation time (min)	—	7.42 ± 0.14^b^	9.55 ± 0.17a	9.77 ± 0.14a
Oil binding capacity (%)	—	78.35 ± 1.41^a^	73.17 ± 0.84b	72.57 ± 0.93b
Centrifuge stability	—	Stable	Stable	Stable
Firmness (N)	—	4.37 ± 0.21^a^	3.77 ± 0.37b	3.53 ± 0.53b
Milting point (°C)	—	55.42 ± 1.11^a^	48.38 ± 0.84b	47.76 ± 0.96b
FFA (as % oleic acid)	2.77 ± 0.25^a^	2.67 ± 0.24^a^	2.68 ± 0.28a	2.71 ± 0.31a
PV (meq O_2_/kg sample)	3.51 ± 0.33^a^	3.44 ± 0.24^a^	3.69 ± 0.16a	3.72 ± 0.17a
TPC (mg GAE/g)	3.95 ± 0.23^c^	4.46 ± 0.13^b^	4.89 ± 0.27b	5.62 ± 0.34a
DPPH (%)	28.15 ± 0.23^c^	30.22 ± 0.16^b^	31.52 ± 0.47b	39.73 ± 0.27a
L*	—	54.36 ± 0.53^b^	56.38 ± 0.62a	52.17 ± 0.46c
a*	—	−1.20 ± 0.14^a^	−1.48 ± 0.19a	−1.51 ± 0.25a
b*	—	2.17 ± 0.14^b^	2.24 ± 0.36b	2.93 ± 0.29a
WI_CIE	—	54.29 ± 0.89b	56.30 ± 0.75a	52.06 ± 0.77b
YI_ (Lab)	—	5.70 ± 0.25b	5.68 ± 0.41b	8.02 ± 0.67a

*Note:* Values are means ± SD. Means having the different case letter (s) within a row are significantly different at *p* ≤ 0.05.

Abbreviations: BGL = bigel without onion phenolic extract, BGLX = bigel with onion phenolic extract, OLGL = oleogel.

#### 3.1.1 Gelation Time

Gelation time. Relative to OLGL (7.42 min), gelation time increased for BGL (9.55 min), consistent with the added aqueous network in the bigel that slows network formation (water‐mediated plasticization and delayed crystallite percolation) (Shakeel et al. 2019). BGL vs. BGLX were not different (9.55 vs. 9.77 min; *p* > 0.05), indicating that the onion phenolic extract did not measurably delay gel setting at the tested load; any small numerical increase in BGLX is within experimental variability rather than a reproducible extract effect.

#### 3.1.2 Oil Binding Capacity (OBC)

Relative to OLGL (78.35%), OBC decreased in the bigels—BGL 73.17% and BGLX 72.57%—consistent with the less rigid dual‐network of bigels, where interactions between hydrophilic and lipophilic phases can slightly reduce oil entrapment (Jandera 2011; Habibi et al. 2022). The small BGL → BGLX difference (73.17% → 72.57%) was not statistically significant (*p* > 0.05), so we do not ascribe OBC changes to the onion phenolic extract at this loading.

#### 3.1.3 Centrifuge Stability, Firmness, and Melting Point

All samples (OLGL, BGL, and BGLX) were stable under centrifugation, indicating robust gel structures. That might be due to the beeswax network in OLGL and bigels preventing phase separation. Firmness was highest in the oleogel (OLGL, 4.37 N) and decreased in the bigels, with values of 3.77 N (BGL) and 3.53 N (BGLX). This reduction in firmness was caused by the incorporation of water in bigels that softened the gel structure and formed a more flexible gel structure, which incorporates both hydrophilic and lipophilic phases, leading to reduced mechanical strength (Chao et al. 2025).

The reduction in firmness due to phenolic extracts is attributed to their interaction with cell wall components, enzymatic activity modulation, and oxidative cross‐linking. Phenolics can bind to polysaccharides like pectin and cellulose, altering structural integrity, while also enhancing cell wall‐degrading enzymes such as polygalacturonases, leading to softening. Additionally, oxidative cross‐linking may disrupt polymer organization, further reducing firmness (Liu et al. 2020; Prodpran et al. 2012). The melting point was 55.42°C in the oleogel (OLGL) and was lower in the bigel systems, at 48.38°C (BGL) and 47.76°C (BGLX). The incorporation of aqueous phases or phenolic compounds lowers the thermal stability of oleogels by disrupting the gelator network, leading to reduced gel strength. Aqueous phases cause phase separation and weaken the cohesive structure, while phenolic compounds interfere with gelation and crystalline behavior, preventing a stable gel matrix. These factors collectively reduce thermal stability (Marangoni and Garti 2018; Pinto et al. 2021).

#### 3.1.4 Free Fatty Acids (FFA) and Peroxide Value (PV)

The FFA and PV values were comparable across all samples, indicating stable oxidative properties. FFA values were similar across all samples, ranging from 2.67% in OLGL to 2.79% in BGLX. Peroxide values were also comparable, with OLGL at 3.72 meq O_2_/kg and the bigels ranging between 3.69 and 3.77 meq O_2_/kg, but these values remained within acceptable limits, indicating good oxidative stability (Table 1). A previous study by Kanelaki et al. (2022) explored the use of rosemary extract (rich in phenolic compounds) in hydrogel, oleogel, and bigel coatings for sardine fillets. The incorporation of rosemary extract significantly retarded lipid oxidation during cold storage, enhancing the preservation of the fish. Also, Baltuonytė et al. (2022) developed bigel‐based spreads enriched with lingonberry pomace, which is high in phenolic content. The addition of the pomace not only provided dietary fiber but also reduced oxidation in the spreads, attributed to the antioxidant activity of the phenolic compounds. Other trends were observed by Hwang (2020); Meng et al. (2024), where the incorporation of bioactive compounds and hydrophilic phases into oleogels resulted in reduced firmness and melting points, as well as slight changes in oil binding and oxidative stability.

Peroxide value and FFA remained low and within commonly accepted limits for baked biscuits throughout the storage period for all formulations, indicating that the lipid system was intrinsically quite stable under the applied conditions. The use of structured lipids (sunflower oil oleogel and bigel) combined with relatively mild storage favored oxidative stability, so that only modest numerical differences were observed between control and extract‐enriched samples. In particular, biscuits containing the onion‐extract bigel tended to show slightly lower PV and FFA values, but these differences were not always pronounced, which is consistent with a system that is already far from the onset of advanced lipid oxidation. The microstructure and spatial distribution of the antioxidant within the bigel matrix likely help to explain these limited effects. In the present bigel system, the beeswax‐structured sunflower oleogel forms the continuous lipid network, while the gelatin–glycerol hydrogel containing the onion extract is present as dispersed aqueous domains. This arrangement can contribute to oxidation control by (i) physically restricting oxygen diffusion and lipid mobility in the structured oil phase and (ii) localizing the hydrophilic phenolic compounds mainly in the aqueous droplets and at the oil–water interface rather than homogeneously dissolved in the bulk fat. Under these conditions, the extract is expected to be more effective in scavenging radicals and transition metals at or near the interface than in markedly lowering bulk PV and FFA values, especially over a limited storage period. Consequently, the overall oxidative status remained acceptable for all treatments, with the onion extract providing an additional, but not dramatic, protective effect within an already stable system.

#### 3.1.5 Total Phenolic and DPPH Radical Scavenging Activity of Sunflower‐Oil Oleogel and Bigel Systems

The antioxidant properties of OLGL, BGL, and BGLX showed incremental improvements in both total phenolic content (TPC) and DPPH radical scavenging activity, especially with the addition of phenolic compounds (Table 1).

The TPC values showed a gradual increase across the different formulations. Total phenolic content was lowest in sunflower oil (3.95 mg GAE/g), increased in OLGL (4.46 mg GAE/g), and was higher in the bigels, reaching 4.89 mg GAE/g in BGL and 5.62 mg GAE/g in BGLX. The higher total phenolic content observed in oleogels and bigel systems, compared to sunflower oil alone, can be attributed to the incorporation of beeswax, which is known to contain phenolic compounds. Studies have reported that beeswax contains phenolic compounds, contributing to its antioxidant properties (Sawicki et al. 2022). The addition of phenolic extract significantly enhanced the TPC, as phenolic compounds, such as those extracted from natural sources like onion or other plant extracts, are rich in antioxidants (Oroian and Escriche 2015).

It is acknowledged that the extraction procedure may result in partial degradation or loss of phenolic compounds. Nonetheless, the method was used uniformly across all formulations to provide relative comparisons of TPC and antioxidant activity, which were sufficient for assessing the functional impact of phenolic enrichment in the gel systems.

This trend aligned with studies by Ferdaus et al. (2025), where the addition of natural antioxidants like phenolic extracts to oleogels increased their phenolic content and antioxidant potential. The increased phenolic content in bigel systems, especially when phenolic extracts are added, highlighted their potential as carriers for bioactive compounds in food systems.

DPPH radical scavenging activity was 28.15% in sunflower oil, increased to 30.22% in OLGL, and was higher in the bigels, with 31.52% in BGL and 39.73% in BGLX (Table 1). The significant increase in DPPH scavenging activity in the bigel with phenolic extract was due to the additional antioxidant capacity provided by the phenolic compounds. This suggested that the incorporation of these bioactive compounds effectively boosted the radical scavenging activity of the system, similar to the results reported by Jeong et al. (2021) who investigated the addition of β‐carotene to oleogels structured with candelilla wax and glycerol monostearate. The results demonstrated that oleogels containing β‐carotene exhibited significantly lower peroxide values, indicating enhanced oxidative stability. This improvement was attributed to the antioxidant properties of β‐carotene. Also, Millao et al. (2025) investigated the addition of β‐carotene to oleogels structured with candelilla wax and glycerol monostearate. The results demonstrated that oleogels containing β‐carotene exhibited significantly lower peroxide values, indicating enhanced oxidative stability. This improvement was attributed to the antioxidant properties of β‐carotene.

The addition of phenolic extracts to sunflower oil‐based bigels significantly improved both TPC and DPPH radical scavenging activity, making these formulations potential candidates for applications requiring enhanced antioxidant functionality.

#### 3.1.6 Color Properties of Sunflower‐Oil Oleogel and Bigel Systems

The color properties showed variations in *L*, *a*, and *b* values between samples, indicating the effects of gelation and the addition of phenolic extracts on the visual appearance of the formulations (Table 1).

Compared with OLGL (*L* = 54.36), BGLX showed a slightly lower lightness (*L* = 52.17; Δ = −3.45 units), and this difference was statistically significant (*p* > 0.05). This reduction in lightness could be attributed to the phenolic compounds presented in the extract, which are known to contain natural pigments that can darken the color of food systems (Cheynier 2012). This observation was consistent with findings by Butkeviciute et al. (2022); Vitolina et al. (2023), where the incorporation of phenolic extracts in oleogel systems led to reduced lightness. For *a* value, which measures the red‐green spectrum, no significant differences were observed between samples. The *b* value, representing the yellow‐blue axis, near results were reported for OLGL and BGL (2.17 and 2.24, respectively), while the *b* value increased in BGLX reached about (2.93). The addition of the phenolic extract increased the *b* value to 2.93, indicating an increase in yellowish hues.

Relative to OLGL, BGL showed a slightly higher whiteness (56.30 vs. 54.29), whereas BGLX exhibited lower whiteness (52.06) and a markedly higher yellowness (8.02 vs. 5.70–5.68), consistent with its elevated b* and the yellow pigments from the onion extract.

#### 3.1.7 Texture Properties of Sunflower‐Oil Olegel and Bigel Systems

The provided data compares the texture properties of sunflower oil–beeswax oleogels and bigels, with and without phenolic extract addition. The parameters assessed include hardness, adhesiveness, cohesiveness, and springiness (Table 2).

**TABLE 2:** Texture properties of sunflower oil‐beeswax oleogel and bigel.

Sample	Texture properties			
	Hardness (mj) cycle 1	Adhesiveness (mj)	Cohesiveness	Springiness (mm)
OLGL	1.10 ± 0.01a	0.60 ± 0.03a	0.36 ± 0.06b	2.84 ± 0.36b
BGL	1.00 ± 0.02b	0.50 ± 0.02b	0.48 ± 0.03a	3.81 ± 0.28a
BGLX	1.00 ± 0.01b	0.51 ± 0.04b	0.48 ± 0.02a	3.76 ± 0.19a

*Note:* Values are means ± SD. Means having the different case letter (s) within a column are significantly different at *p* ≤ 0.05.

Abbreviations: BGL = bigel without onion phenolic extract, BGLX = bigel with onion phenolic extract, OLGL = oleogel.

OLGL exhibits a hardness of 1.10 mJ, while both bigel samples (BGL and BGLX) show a slightly lower hardness of 1.00 and 1.00 mJ, respectively. This reduction in hardness upon transitioning from oleogel to bigel could be attributed to the incorporation of the hydrogel phase, which may disrupt the crystalline network of the oleogel, leading to a softer structure. This observation aligns with findings that the addition of hydrophilic components can modulate the firmness of gelled oil systems (Baltuonytė et al. 2022).

OLGL has an adhesiveness of 0.60 mJ, whereas BGL and BGLX display lower adhesiveness values of 0.50 and 0.51 mJ. The decrease in adhesiveness in bigels may result from the presence of the hydrogel phase, which can alter the surface properties and reduce stickiness. The slight increase in adhesiveness upon adding phenolic extract is minimal, suggesting that the extract does not significantly impact this property.

Cohesiveness measures the internal bonding strength of the gel matrix. OLGL shows a cohesiveness of 0.36, while both BGL and BGLX samples exhibit higher cohesiveness values of 0.48 and 0.48, respectively. The enhanced cohesiveness in bigels could be due to the formation of a more interconnected network structure resulting from the combination of hydrogel and oleogel phases. This synergistic interaction can lead to a more resilient gel matrix (Khelifi et al. 2020).

Springiness refers to the ability of the gel to recover its original shape after deformation. The oleogel has a springiness of 2.84 mm, whereas the bigels demonstrate higher springiness values of 3.81 and 3.76 mm. The increased springiness in bigels may be attributed to the elastic properties imparted by the hydrogel phase, which enhances the gel's ability to recover after compression.

The addition of phenolic extract to the bigel formulation does not appear to significantly alter the texture properties compared to the bigel without the extract. This suggests that the phenolic extract, at the concentration used, does not markedly influence the structural characteristics of the bigel.

From OLGL to BGL, hardness and adhesiveness decreased, while cohesiveness and springiness increased (all *p* < 0.05), indicating a softer, more elastic bigel. By comparing between BGL and BGLX, differences were small and not significant (*p* > 0.05), so the extract did not materially alter gel texture at this dose.

#### 3.1.8 Viscosity of Sunflower‐Oil Olegel and Bigel Systems

**FIGURE 2:**
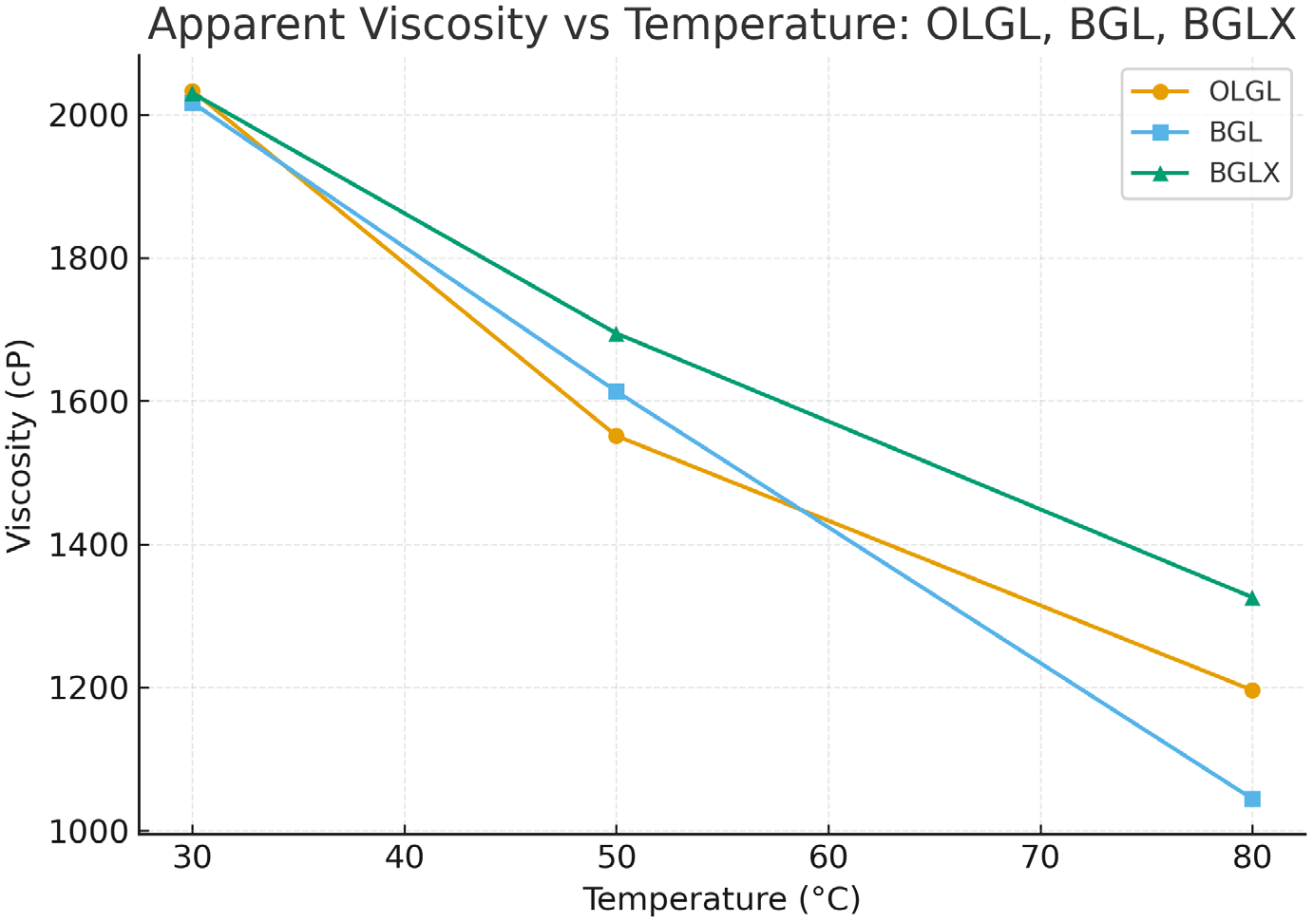
Viscosity of sunflower oil–beeswax oleogel (OLGL) and bigels (BGL, BGLX). BGL = bigel without onion phenolic extract, BGLX = bigel with onion phenolic extract, OLGL = oleogel.

The viscosity values of OLGL, BGL, and BGLX at different temperatures (30°C, 50°C, and 80°C) demonstrated significant trends influenced by temperature and the composition of the gels (Figure 2). Viscosity is a critical property in determining the flow behavior of gels, which directly impacts their applicability in food systems such as spreads, baked goods, or emulsions.

At 30°C, all samples showed high viscosity, with BGL and BGLX displaying values close to those of OLGL (around 2016.67–2033.33 cP). This suggested that at lower temperatures, all three systems exhibited similar structural integrity and resistance to flow. The high viscosity at this temperature could be attributed to the solidified state of the beeswax and the gel matrix, which restricted movement within the gel network.

As the temperature raised to 50°C, the viscosity of all the samples decreases, as expected, due to the thermal softening of the gel network. BGLX maintained a higher viscosity (1694.44 cP) compared to both the OLGL (1551.28 cP) and BGL (1613.33 cP). This behavior may be due to the addition of phenolic extract which increased the thermal stability of the gel, preventing a significant drop in viscosity as the temperature increased. The phenolic compounds likely enhanced the gel network's stability by forming additional hydrogen bonds or hydrophobic interactions, which resist the thermal disruption of the gel matrix (Feng et al. 2023; Schefer et al. 2021). Previous research by Ferdaus et al. (2025); Gonçalves et al. (2024) has shown that the incorporation of bioactive compounds can increase the thermal stability of oleogel and bigel systems, resulting in a slower decline in viscosity with rising temperatures.

At 80°C, all samples displayed a further reduction in viscosity due to the melting of the beeswax and weakening of the gel matrix. However, BGLX exhibited a notably higher viscosity (1325.85 cP) compared to BGL (1044.44 cP) and OLGL (1196.15 cP). The higher viscosity in the phenolic extract sample could be attributed to the phenolic compounds forming a more robust gel matrix that resisted the heat‐induced breakdown. Ferdaus et al. (2025); Kwon and Chang (2022) observed similar behavior in oleogels and bigels enriched with bioactive compounds, where the inclusion of phenolics led to better heat stability and higher viscosity at elevated temperatures compared to control samples.

The incorporation of phenolic extract into sunflower bigel resulted in increased viscosity at all temperatures, suggesting that phenolics enhance the gel network's strength and thermal stability. This can be beneficial in applications where temperature fluctuations are common, as the phenolic‐enriched bigel maintains a more stable viscosity, which is crucial for the texture and flow behavior of food products.

#### 3.1.9 AFM of Sunflower‐Oil Olegel and Bigel Systems

**FIGURE 3:**
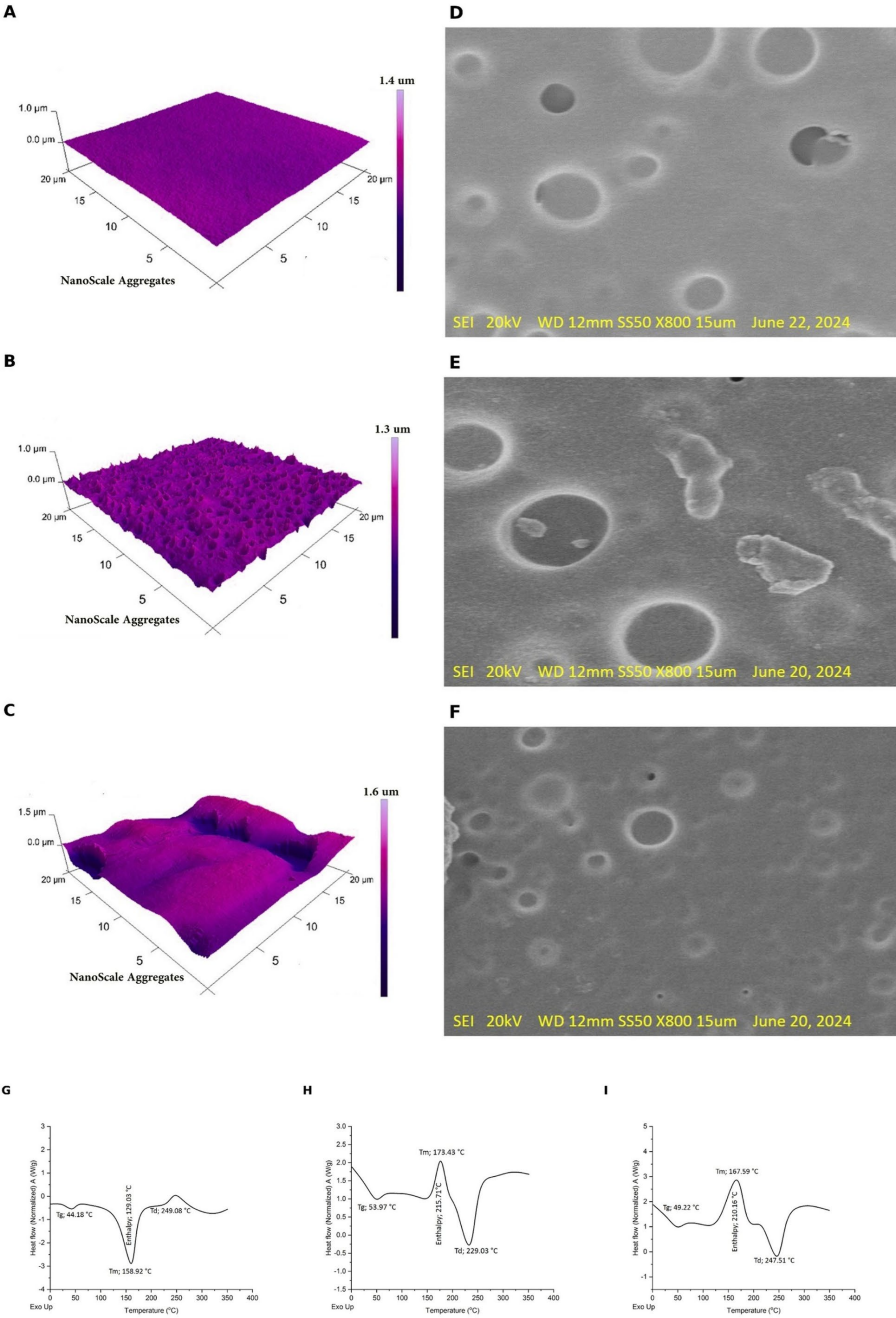
Microstructural and thermal analysis of different gel formulations. A = AFM for Oleogel, B = AFM for Bigel, C = AFM for Bigel with onion phenolic extract, D = SEM for Oleogel, E = SEM for Bigel, F = SEM for Bigel with onion phenolic extract, G = DSC for Oleogel, H = DSC for Bigel, I = DSC for Bigel with onion phenolic extract.

The Atomic Force Microscopy (AFM) images provided for OLGL, BGL, and BGLX showed distinct differences in their surface morphology and nanoscale structure, which could be attributed to the interaction between the components in each gel type (Figure 3). AFM scans were acquired over 20 × 20 μm areas. From the z‐scale bars, the out‐of‐plane height span within the frame was ~1.0–1.4 μm for OLGL, ~1.0–1.3 μm for BGL, and ~1.1–1.6 μm for BGLX, indicating a higher peak‐to‐valley amplitude in the phenolic‐enriched bigel.

The surface of OLGL exhibited a smooth appearance, as shown in Figure 3A. The presence of an even texture and nanoscale aggregates suggested a uniform distribution of oil and beeswax, contributing to the formation of a stable gel matrix. The smooth surface was typical for oleogels, which rely on the structuring ability of waxes to trap the oil phase. This has been reported in other studies, such as those by Wang et al. (2023), who also observed smooth surfaces in sunflower oil–beeswax gels, contributing to good spreadability and mouthfeel in food applications.

AFM image for BGL (Figure 3B) showed more roughness with distinct peaks, indicating the interaction between the oleogel and the hydrogel. This structure suggested that the hydrogel, which provided a water phase, introduced some irregularity in the otherwise smooth surface of the oleogel. The rough surface was a sign of phase aggregation between the hydrophobic and hydrophilic components of the bigel, a phenomenon reported in Chao et al. (2024), who noted similar surface disruptions in bigels composed of oil and water phases. This morphology might influence the texture and release properties in food and cosmetic formulations, making the bigel more suitable for applications requiring slow release or enhanced mechanical properties.

The third AFM image (BGLX) (Figure 3C) showed even more pronounced roughness and larger nanoscale aggregates. The addition of the onion phenolic extract likely contributed to this increased roughness, as polyphenols could interact with the gel matrix, forming more complex structures. This is consistent with findings by Chao et al. (2025), who observed that polyphenolic compounds can cause increased surface roughness and aggregation in gel matrices. The increased roughness may also contribute to enhanced antioxidant properties, as the phenolic compounds are more available for interaction with free radicals (Brito et al. 2021).

In conclusion, the AFM images revealed significant changes in the surface morphology as the formulation progresses from oleogel to bigel and bigel with phenolic extract. These changes directly affected the physical properties such as texture, mechanical strength, and antioxidant potential.

#### 3.1.10 SEM of Sunflower‐Oil Olegel and Bigel Systems

The SEM images showed structural differences among the oleogel and bigel systems (Figure 3D–F). All micrographs were collected at 800× with a 15 μm scale bar (SEI, 20 kV, WD = 12 mm). Based on this scale, the circular pores/voids visible in OLGL typically spanned ~2–6 μm in equivalent diameter; BGL exhibited a broader distribution of ~3–12 μm with occasional larger coalesced features; BGLX presented a mixed population with many small pores (~1–4 μm) superimposed on 4–10 μm voids. In the first SEM image (OLGL) (Figure 3D), the oleogel prepared from beeswax and sunflower oil showed a smooth and homogenous surface. The appearance of spherical voids or pores could be attributed to the beeswax crystals dispersed within the sunflower oil matrix. The size and distribution of these voids affect the mechanical properties and stability of the oleogel. Previous studies, such as those by Pang et al. (2020), suggest that oleogels stabilized with beeswax show crystalline structures, which enhance the firmness and oil‐binding capacity of the material. The relatively uniform structure seen here supported the enhanced oil‐holding and textural properties of the oleogel.

The second image of the bigel (BGL) (Figure 3E), prepared by combining oleogel and hydrogel, showed more complex and heterogeneous features. The voids or pores appeared larger and irregular, which could be due to the integration of the hydrogel phase into the oleogel matrix. This structure likely enhanced water‐binding properties while slightly reducing the firmness of the gel, as supported by studies like Zhang et al. (2025), who indicated that the incorporation of hydrogels into oleogels increased hydration but altered the structural integrity. The rougher and more porous structure was in line with a softer texture, as noted in the properties of bigels.

The third image (BGLX) (Figure 3F) showed an even more disrupted surface morphology compared to BGL. The presence of onion phenolic compounds might interfere with the gel matrix, creating aggregated particles and irregularities in the surface. These phenolic compounds may act as antioxidants while affecting the gelation process and overall structure. This is consistent with findings by Ćorković et al. (2021), where the incorporation of phenolic compounds into gel systems disrupted the gel matrix, leading to more heterogeneous structures. The presence of phenolic extract could also influence the antioxidant properties and enhance the shelf‐life of the final product. These size ranges support the textural trends (lower firmness from OLGL to BGL) and the AFM observation of increased surface heterogeneity in the bigel systems.

#### 3.1.11 DSC of Sunflower‐Oil Olegel and Bigel Systems

For the thermal properties as shown in the DSC images, each formulation presented distinct thermal behavior, as indicated by the glass transition temperature (Tg), melting temperature (Tm), and degradation temperature (Td) (Figure 3).

OLGL (Figure 3G) exhibited a glass transition temperature (Tg) of 44.18°C, a melting temperature (Tm) of 158.92°C, and a degradation temperature (Td) of 249.08°C. The Tg observed here was lower than that of bigel formulations, likely due to the homogeneous structure of the oleogel, which was primarily composed of lipid components. Consistent with prior work, beeswax crystallizes in sunflower oil to form a three‐dimensional crystal network that immobilizes the liquid phase, as evidenced by microscopy and DSC signatures characteristic of wax‐based oleogels (Pang et al. 2020).

BGL (Figure 3H), combining the oleogel and hydrogel, showed a higher Tg (53.97°C) compared to the oleogel. The increase in Tg suggested enhanced structural rigidity, likely due to the interaction between the hydrogel's water‐based phase and the lipid matrix. A similar phenomenon was reported by Drabik et al. (2020), who reported that the mechanical properties of lipid bilayers composed of different phospholipids found that the bending rigidity coefficient decreased with an increase in the transition temperature of the lipids. This suggests that modifications affecting the lipid composition and interactions can alter the mechanical rigidity and phase behavior of the bilayer. The melting temperature (Tm) raised to 173.43°C, and the enthalpy was higher (215.71 J/g), indicating more energy was required to melt the system, likely due to the enhanced network formed between the oleogel and hydrogel phases. The Td was lower (229.03°C), suggesting the bigel degraded earlier than the oleogel, possibly due to the presence of the aqueous phase contributing to earlier degradation.

The addition of phenolic extract from onion further modified the thermal properties (Figure 3I). The Tg was 49.22°C, slightly lower than that of the bigel without phenolic extract, reducing the overall rigidity of the system. This is consistent with findings by Garcia‐Carrasco et al. (2022), where the incorporation of phenolic compounds in lipid‐based systems decreased Tg due to their interaction with the matrix, softening the network. The melting temperature (Tm) was slightly lower (167.59°C) than the bigel without the extract, which could be attributed to the phenolic extract disrupting the lipid‐crystal network. However, the Td (247.51°C) was higher than the regular bigel, indicating the phenolic extract improved the thermal stability of the system. This aligns with the research by Mallegni et al. (2025), which demonstrated that the role of natural antioxidants, particularly phenolic compounds, in stabilizing biodegradable polymers. The incorporation of these compounds into biopolymer matrices was shown to improve resistance to thermal and oxidative degradation. The phenolic compounds act by scavenging free radicals and decomposing peroxides, thereby enhancing the thermal stability of the biopolymers.

### 3.2 Effect of Using Oleogel and Bigel as a Fat Replacer in Biscuits Preparation

#### 3.2.1 Chemical Composition of Biscuits Prepared From OLGL, BGL and BGLX

The proximate analysis of the biscuits revealed significant differences in moisture and fat content, while protein, ash, and carbohydrate levels remained relatively stable (Table 3). Moisture was lowest in control biscuits (4.53%) and higher in those made with structured lipids, with values of 4.92% in OLGL, 5.34% in BGL, and highest in BGLX (5.78%), likely due to the hydrogel phase, which enhances water retention by forming a three‐dimensional network that traps moisture (Chao et al. 2024). Relative to control biscuits, OLGL, BGL, and BGLX showed higher fat fractions (22.03%, 21.52%, and 21.34%, respectively) and slightly lower carbohydrate by difference, consistent with the structured‐lipid substitution and higher moisture retention. In contrast, protein and ash did not differ appreciably among formulations (*p* > 0.05). Thus, the reformulated biscuits exhibit a shifted macronutrient profile—higher fat and lower carbohydrate—while protein and ash remain stable. These findings align with previous studies demonstrating that structured lipid‐based systems like oleogels and bigels modify food matrix properties by influencing moisture, fat retention, and textural attributes, making them viable functional fat replacers in bakery applications (Perța‐Crișan et al. 2023). Oleogels can stabilize air bubbles in the batter, leading to a different fat distribution and potentially higher measured fat content. Studies have shown that the use of oleogels in bakery products can influence the fat content and distribution within the product (da Silva, Dos Santos, et al. 2023).

**TABLE 3:** Chemical composition of prepared biscuits.

Parameter	Control	OLGL	BGL	BGLX
Moisture (%)	4.53 ± 0.22d	5.07 ± 0.22c	5.53 ± 0.08b	5.78 ± 0.16a
Fat (%)	20.04 ± 0.51c	22.03 ± 0.42a	21.52 ± 0.41b	21.34 ± 0.33b
Protein (%)	9.82 ± 0.19a	9.76 ± 0.11a	9.63 ± 0.22a	9.56 ± 0.21a
Ash (%)	2.01 ± 0.11a	2.15 ± 0.12a	2.20 ± 0.11a	2.33 ± 0.10a
Carbohydrates (%)	63.60 ± 0.54	60.99 ± 0.43	61.12 ± 0.45	60.99 ± 0.37

*Note:* Values are means ± SD. Means having the different case letter (s) within a row are significantly different at *p* ≤ 0.05.

Abbreviations: BGL = Biscuits prepared with bigel, BGLX = Biscuits prepared with bigel contains onion phenolic extract, OLGL = Biscuits prepared with oleogel.

#### 3.2.2 Color and Antioxidant Properties of Biscuits Prepared From OLGL, BGL and BGLX

**FIGURE 4:**
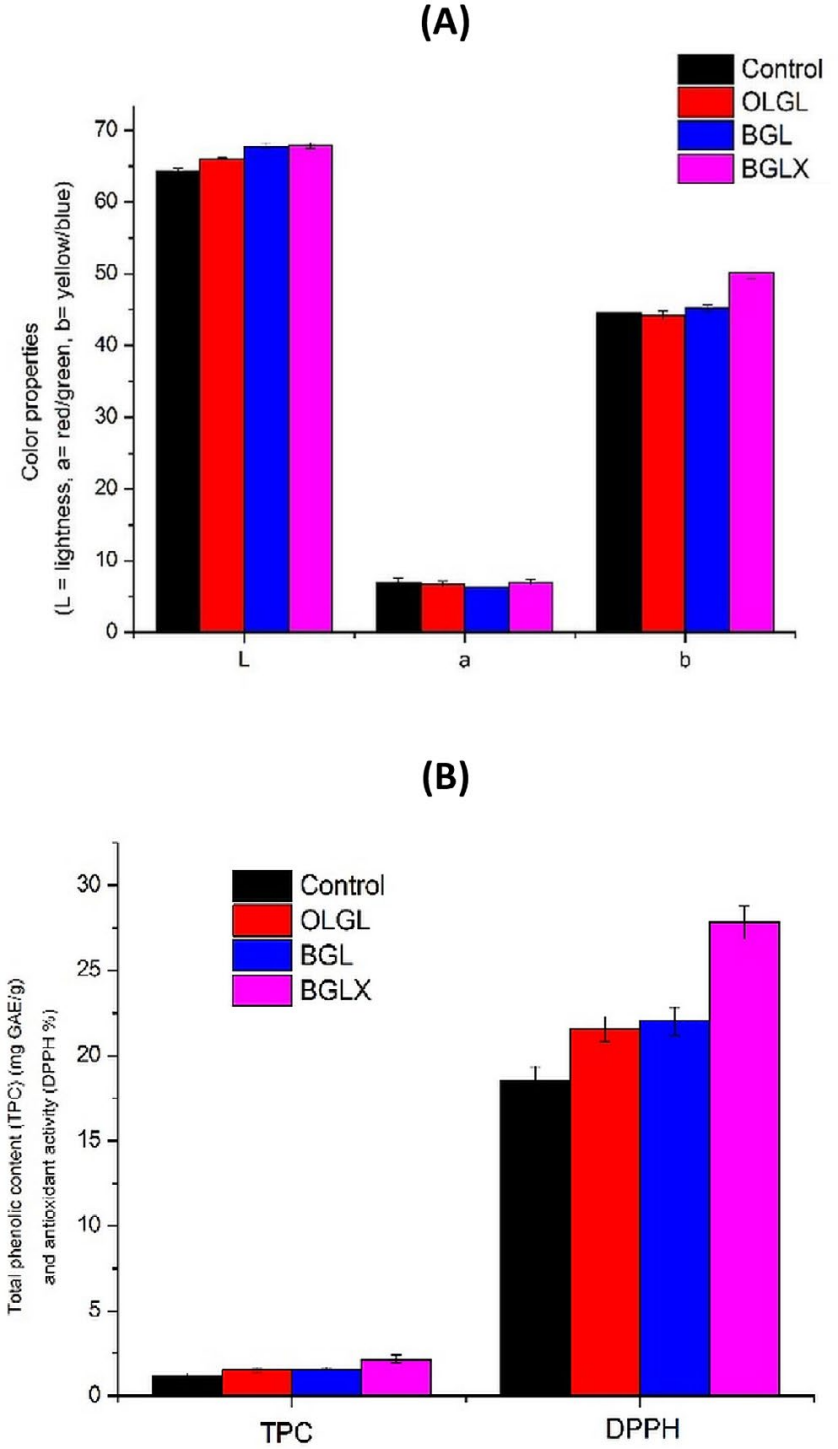
Color properties (A), total phenolic content (TPC) and antioxidant activity (DPPH %) (B) of prepared biscuits. BGL = Biscuits prepared with bigel containing onion phenolic extract, OLGL = Biscuits prepared with oleogel. Values are means ± SD. Means having the different case letter(s) within a row are significantly different at *p* ≤ 0.05.

Color is the first sensory characteristic that affects the appearance of food and consumer acceptance of food (Mbassi et al. 2022). The data provided in the Figure 4 showed the color properties with the parameters Lightness (L*), Redness/Greenness (a*), and Yellowness/Blueness (b*), for control biscuits, OLGL (Biscuits prepared with oleogel), BGL (Biscuits prepared with bigel), and BGLX (Biscuits prepared with bigel contains onion phenolic extract). The Control biscuits exhibited the significantly lowest L value (64.21) as compared to the biscuits formulated with OLGL, BGL, and BGLX. The lower L value (lightness) observed in control biscuits (64.21) compared to those formulated with oleogels and bigels can be attributed to increased Maillard browning and caramelization during baking. Conventional fats in control biscuits may promote these reactions, leading to a darker color. In contrast, oleogels and bigels, which often contain structured oils and possibly phenolic compounds, can reduce browning due to their antioxidant properties and different heat transfer characteristics, resulting in a lighter color. Studies have shown that biscuits made with oleogels exhibit higher lightness values, indicating less browning compared to those made with traditional fats (Mata‐Mota et al. 2023; Onacik‐Gür and Żbikowska 2020). All remaining samples had progressively higher *L* values of 66.06, 67.82, and 67.86, respectively. Moreover, the BGL Biscuit and BGLX Biscuit did not show significant change in L value.

As for values, all biscuit samples were very close and did not show significant differences between them, with values ranging from 6.34 for BGL biscuits to 7.01 for Control and BGLX biscuits which had the same value.

On the other hand, b value of BGLX biscuits appeared with a significantly higher mean 50.15 as compared with the other biscuit samples while control (44.54), OLGL (44.26), and BGL (45.22) biscuits were not significantly different between them. This significant increase in the yellow component in BGLX biscuits can be attributed to the presence of yellow pigments in the onion phenolic extract. The results were in agreement with Gulati et al. (2023) and Kumar et al. (2022) whose reported onion peel extract considered a rich source for red and yellow pigment. The results indicate that utilizing these novel fat substitutes can lead to improved color properties in biscuits, potentially enhancing their marketability while offering healthier options by replacing traditional fats.

#### 3.2.3 Total Phenolic Content (TPC) and Antioxidant Activity (DPPH %) of Biscuits Prepared From OLGL, BGL and BGLX

As shown in Figure 4A,B, TPC and DPPH (%) are gradually significantly (*p* ≤ 0.05) increased by the extent of onion phenolic extract. The results indicate that incorporating oleogel (OLGL), bigel (BGL), and bigel with onion phenolic extract (BGLX) significantly enhanced Total Phenolic Content (TPC) and DPPH radical scavenging activity in biscuits compared to the control biscuits (*p* ≤ 0.05). The control biscuits exhibited the lowest TPC (1.22 mg GAE/g) and DPPH activity (18.53%), likely due to the absence of structured lipid matrices or bioactive compounds.

The use of oleogel (OLGL) and bigel (BGL) resulted in a moderate increase in TPC (1.53–1.61 mg GAE/g) and DPPH activity (21.57%–22.03%), which can be attributed to the retention of bioactive compounds during baking. Oleogels and bigels, being structured lipid systems, may reduce oxidation and degradation of inherent phenolics in the biscuit matrix. Studies have shown that oleogel and bigel matrices can encapsulate and protect phenolic compounds, thereby preserving their bioactivity and increasing antioxidant potential (de Oliveira 2021).

The BGLX biscuits exhibited the highest TPC (2.17 mg GAE/g) and DPPH activity (27.83%), significantly surpassing all other formulations (*p* ≤ 0.05). This increase is directly attributed to the incorporation of onion phenolic extract, which contains flavonoids (e.g., quercetin), organosulfur compounds, and phenolic acids known for their strong antioxidant activity (Pérez‐Gregorio et al. 2014). The onion‐derived phenolics not only contributed directly to the increased TPC but also enhanced DPPH radical scavenging capacity, confirming their role in improving oxidative stability.

### 3.3 Effect of Storage on Biscuits Prepared From OLGL, BGL and BGLX

#### 3.3.1 Moisture Content of Prepared Biscuits

The changes in the biscuits moisture level during the storage display in Figure 4 which affected by the type of addition. The results indicate that biscuits formulated with bigel (BGL) and bigel enriched with onion phenolic extract (BGLX) retained significantly higher moisture content than oleogel (OLGL) and control biscuits throughout the three‐month storage period (*p* ≤ 0.05). The BGLX biscuits exhibited the highest moisture retention, likely due to the hygroscopic nature of phenolic compounds, which interact with water molecules, forming additional hydrogen bonds and reducing evaporation. Studies have shown that phenolic‐rich extracts improve water‐holding capacity by forming cross‐linkages with biopolymers in the food matrix, leading to enhanced moisture stability (Khalil et al. 2023). Additionally, the dual‐phase hydrogel‐organogel structure in bigels contributes to superior water retention compared to oleogels, as hydrophilic domains trap more moisture and slow down water migration.

Over time, all biscuits experienced moisture loss, with control biscuits showing the most significant reduction (from 4.53% to 3.88%), likely due to the absence of structured lipid matrices. Oleogel‐based biscuits (OLGL) retained more moisture than the control but were less effective than bigel formulations, as oleogels primarily restrict oil migration rather than retain water. In contrast, bigels, particularly BGLX, slowed moisture loss, confirming findings that bigel‐containing foods exhibit lower water activity and enhanced shelf‐life stability (Francavilla et al. 2023). These findings suggest that bigels, especially those enriched with phenolic compounds, are promising fat replacers in bakery products, offering enhanced moisture retention, improved texture, and prolonged freshness compared to traditional fats.

The moisture content typically decreases due to the hygroscopic nature of dried products, which absorb water from their surroundings. Factors such as environmental conditions specifically temperature and relative humidity (RH) as well as the characteristics of the packaging material play a significant role in determining the amount of water that is taken up (Guiné 2018; Khan et al. 2017).

#### 3.3.2 Hardness of Prepared Biscuits

The results of the texture analysis demonstrate that the addition of bigel, particularly when combined with onion phenolic extract, can significantly reduce the hardness of biscuits over time (Figure 4). This suggests a synergistic interaction between these components, influencing the overall texture of the biscuits.

The findings of this study align with previous research on the impact of ingredients on food texture, particularly in the context of using oleogels as a substitute for saturated fats (da Silva, Dos Santos, et al. 2023; Ghendov‐Mosanu et al. 2023). The control biscuits, lacking any additives, consistently exhibited the highest hardness.

Biscuits prepared with bigel (BGL) exhibited a more pronounced reduction in hardness over time. However, the BGLX biscuits, incorporating both bigel and onion phenolic extract, demonstrated the greatest decrease in hardness. These results are consistent with previous studies that have investigated the effects of bigel and onion phenolic extract on food texture.

Han et al. (2024) found that bigel incorporation significantly enhanced bread texture, resulting in a softer and more pliable product. Similarly, Quilaqueo et al. (2022) demonstrated that bigels can effectively replace traditional fats in cookies without compromising their desired textural properties. This synergistic effect could be attributed to the complementary properties of these components. Prokopov et al. (2018) found that onion phenolic extract can improve bread texture by reducing crumb hardness. This enhancement is attributed to the presence of onion phenolic compounds, which interact with the dough matrix to create a tenderer and desirable bread texture (Bedrníček et al. 2020).

#### 3.3.3 Peroxide Value of Prepared Biscuits

Peroxides are intermediate compounds in the autoxidation reactions. Generally, peroxide value (PV) is the most widely used measurement to estimate the induction oxidation period of the oxidative rancidity, which occurred during storage and is a good criterion for the prediction of the quality and stability of oils. Consequently, oils with a high degree of unsaturation are most susceptible to autoxidation. It is well known that fresh oils have peroxide values below approximately 10 meq O_2_/kg, while oils that have spoiled and become rancid present peroxide values between 20 and 40 meq O_2_/kg (Akubugwo and Ugbogu 2007; Nangbes et al. 2013).

**FIGURE 5:**
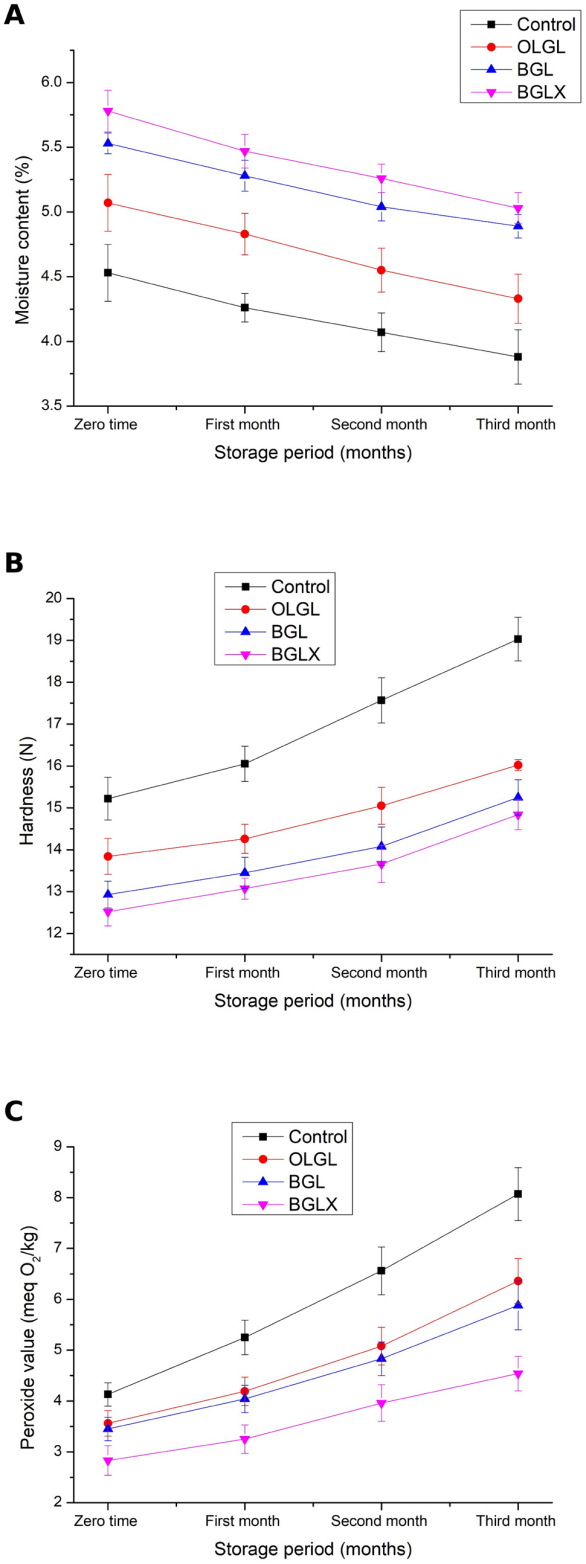
Effect of storage for 3 months on moisture content (A), hardness (B), and peroxide Value (C) of prepared biscuits. BGL = Biscuits prepared with bigel, BGLX = Biscuits prepared with bigel contains onion phenolic extract, OLGL = Biscuits prepared with oleogel. Values are means ± SD. Means having the different case letter(s) within a row are significantly different at *p* ≤ 0.05.

Data in Figure 5 showed that the control biscuits had the highest PV (4.13 meq O_2_/kg) compared to the OLGL and BGL biscuits (3.56 and 3.45 meq O_2_/kg) at zero time, while the lowest PV was in BGLX biscuits (2.83 meq O_2_/kg). The decrease of PV in BGLX biscuits can be explained by their high content of total phenolic compounds (TPC) and its antioxidant activity, as it has the highest content of TPC and antioxidant activity among all treatments. This was in line with the results reported by Martinović et al. (2020), who indicated that an oleogel system with phenolic compounds could inhibit lipid peroxidation more than an oil with the same amount of phenolic compounds. Simultaneously, Hwang (2020) pointed out that firmer gels oxidize slowly because the dense structure reduces the diffusion of oxygen into the volume of the oleogel; this, in turn, slows down the oxidation of the oil. Also, Sobolev et al. (2022) show that oleogels structured with beeswax fractions could be beneficial in fat‐containing products with improved oxidation resistance. In addition, there are no significant differences (*p* ≤ 0.05) between OLGL and BGL biscuits in PV at zero time and during storage (Figure 5).

On the other hand, the longer the storage period, the higher the PV in Biscuits. For example, PV in control biscuits after 3 months is 8.07 meq O_2_/kg decreases significantly (*p* ≤ 0.05), 6.36, 5.88, and 4.54 meq O_2_/kg for OLGL, BGL, and BGLX biscuits, respectively, under the same conditions of storage. Generally, the presence of OLGL, BGL, and BGLX biscuits decreases the concentration of PV during storage compared with control Biscuits. However, the PV values of all the analyzed samples, even after the storage period of 3 months, are below the maximum allowed by international standards of up to 15 meq O_2_/kg oil. This indicates that the process conditions of the oleogel and bigel preparation did not promote primary oxidation.

It could be concluded that the results confirm that sunflower oil‐based bigels, particularly those enriched with phenolic extracts, exhibit superior physicochemical and antioxidant properties compared to oleogels. The addition of a hydrogel phase modified gelation dynamics, reducing firmness and melting point while enhancing cohesiveness and springiness. The incorporation of phenolic compounds significantly increased total phenolic content and antioxidant activity, improving oxidative stability. Storage studies demonstrated the superior moisture retention and reduced lipid oxidation in biscuits formulated with bigels, particularly BGLX. These findings suggest that bigels are promising alternatives to conventional fats, with potential applications in functional food formulations aimed at improving both nutritional and structural properties.

## 4 Conclusion

Sunflower oil–beeswax bigels can replace conventional bakery fats while maintaining functionality and improving oxidative robustness. Relative to oleogels, introducing a hydrogel phase produces the expected softening and modest thermal lowering associated with a biphasic, water‐plasticized network. Phenolic enrichment with a quercetin‐rich onion extract does not materially alter bulk mechanics but clearly enhances antioxidant capacity. As a fat replacer in biscuits, these structured lipids shift product composition toward higher moisture retention without appreciable changes in protein or ash, while supporting stable color and texture. Baseline oxidative status is comparable among structured‐lipid biscuits; during storage, bigels slow peroxide formation, and phenolic enrichment further attenuates oxidation within accepted limits, with whiteness and yellowness indices aligning with instrumental color trends and reflecting the extract's pigments without compromising appearance. Overall, bigels offer a viable, lower‐saturated‐fat route for bakery reformulation, and phenolic‐enriched bigels add a shelf‐life benefit without penalizing texture.

## Author Contributions

**Mahmoud Younis:** supervision, writing – original draft. **Reham Kamel, Mostafa Ali, Said El Harkaoui, Eslam Elshaqwir:** project administration, writing – original draft. **Mohamed El‐Bana**, **Diaeldin Omer Abdelkarim, Mohamed Saleh:** writing – review and editing, supervision, funding acquisition. **Amira H. Sharshar, Amira E. Abd El‐Gwad, Menna Allah M. Youssef**, **Shimaa E. Abd EL‐Hamed, Alshaimaa M. Hamouda, Mahmoud Elsayed, Naglaa K. Beltagy, Tahia R. Al‐Khawaga, Mariam A. El‐Khatib, Nahla A. Felays:** writing – original draft, writing – review and editing. **Mona Hussein Hassan**, **Mohamed Abdelbaset Salama**, **Mohamed Abdin**, **Maha Gomaa:** conceptualization, methodology, formal analysis, investigation, visualization, writing – original draft, writing – review and editing.

## Ethics Statement

The authors have nothing to report.

## Consent

The authors have nothing to report.

## Conflicts of Interest

The authors declare no conflicts of interest.

## Data Availability

The data that support the findings of this study are available from the corresponding author upon reasonable request.
